# Biosurfactants from *Trichoderma* Filamentous Fungi—A Preliminary Study

**DOI:** 10.3390/biom11040519

**Published:** 2021-03-30

**Authors:** Michał Piegza, Joanna Pietrzykowska, Joanna Trojan-Piegza, Wojciech Łaba

**Affiliations:** 1 Department of Biotechnology and Food Microbiology, Wroclaw University of Environmental and Life Sciences, 37 Chelmonskiego Street, 51-630 Wroclaw, Poland; joanna.pietrzykowska@o2.pl (J.P.); wojciech.laba@upwr.edu.pl (W.Ł.); 2 Faculty of Chemistry, University of Wroclaw, 14 F. Joliot-Curie Street, 50-383 Wroclaw, Poland; joanna.trojan-piegza@chem.uni.wroc.pl

**Keywords:** *Trichoderma citrinoviride*, biosurfactants, IR, GC, NMR

## Abstract

Biosurfactants represent a structurally diverse group of secondary metabolites produced by bacteria, yeasts, and filamentous fungi. Their character is often associated with numerous additional properties. The observation of *Trichoderma* fungi of various species used as a source of bioinhibitors against pathogenic plants fungi focuses attention to the often quite specific behavior of preparations in contact with, among others, plant leaves, dependent on strain. Thus, an evaluation of the selected strains belonging to the species: *T. atroviride*, *T. citrinoviride,* *T. reesei* and *T. harzianum* was conducted towards their capability of the extracellular secretion of surfactants, with a simultaneous attempt to pre-determine their chemical nature. Two mineral-organic media were used for this purpose, and the culture fluid was extensively tested using a variety of methods. A decrease in surface tension was observed in culture fluid of each tested strain, especially *T. citrinoviride* HL and C1. The results strongly depended on medium composition, of which Saunders 1 and MGP 1 were most beneficial. The secreted compounds were further analyzed to pre-determine their chemical nature using IR, GC, and NMR. In the case of most efficient biosurfactant producers, a lipopeptide structure of the surfactants was concluded.

## 1. Introduction

Biochemical compounds classified as surfactants possess the ability to reduce surface tension by accumulating on the surface of two immiscible liquids [1]. They are characterized by a specific amphiphilic structure, which incorporates a hydrophilic and hydrophobic region [2]. Conversely, the hydrophobic, non-polar component exhibits high solubility in non-polar liquids, oils, and lack of solubility in water. Most often, it is structurally an aliphatic hydrocarbon chain, either branched, unbranched or composed of an aromatic hydrocarbon with a long hydrocarbon chain [3]. It has been confirmed that biosurfactants tend to improve hydrophilic properties of agricultural soils, therefore they find practical use as additives to fertilizers and pesticides, which results in their enhanced solubility and action. In the food industry, they are applied as food additives, e.g., in the production of sweeteners or in the clarification of juices and beer. They have a beneficial effect on the texture of food products. Cosmetic companies that incorporate biosurfactants in their production confirm their valuable features [4,5]. For several years, research has also been conducted on the antiviral properties of biosurfactants [6].

Filamentous fungi of the genus *Trichoderma* are ubiquitous worldwide in a wide variety of soils. They find multiple applications in diverse industrial sectors due to their capability to produce numerous enzymes, such as cellulases, xylanases, pectinases, β-1,3-glucanases, chitinases, and peptidases. Hence, their application in fodder, food, brewing, bioethanol production, and textile and paper industries is feasible. This type of microorganism mainly supports the growth of plants and protects them against phytopathogens. Some strains are capable of producing siderophores, i.e., low molecular weight iron chelators [7]. However, the potential of fungi of the genus *Trichoderma* in the production of biosurfactants is different. Da Silva et al. [8] mention *Trichoderma* fungi as one of the types of several species as capable biosurfactants or bioemulsifiers biosynthesis but of unknown chemical nature. Sena et al. [9] indicates several *Trichoderma* strains among which there is a certain ability to emulsification, but most give a negative result in the drop collapse test. The hard suggestion about *Trichoderma viride* biosurfactants was indicated by Maheswari and Parveen [10] Thus, at the moment, this topic is still poorly explored. So far, the fact has been confirmed that *Trichoderma reesei* produces hydrophobins, which are low molecular weight proteins with high surface-active and amphiphilic properties. Filamentous fungi biosynthesizes two classes of hydrophobins HFBI and HFBII, where the genes hfb1 and hfb2 play a regulatory role in the synthesis of hydrophobins [11]. Fungal hydrophobins are a group of surfactants, self-organizing proteins. It was found that hydrophobins, especially HFBI, displayed high affinity to surfactants. The reduction of disulphides in the protein resulted in a complete loss of affinity to the surfactant, which confirms that the effect depends on their stabilized conformation. Some of the fungi secrete glycolipid surfactants, but the genetic basis for their production is largely unknown. Lipids of mannosyritrol (MEL) were first isolated from the dimorphic fungus *Ustilago maydis,* and also later detected in *Candida antarctica* and *Geotrichum candidum.* In addition, sophorolipids are secreted by *Candida bombicola* [11], as well as other representatives of the genus *Candida*, or other fungi [12]. Bhardwaj et al. [12] suggests that most fungal biosurfactants are lipid derivatives and often constitute a protein-lipid-carbohydrate complex. World reports indicate that bacteria, in particular from the genus *Bacillus* and *Pseudomonas*, are most frequently used for the production of biosurfactants. Little is known about this potential found in yeast and especially filamentous fungi [8].

In this study, the potential of selected filamentous fungi of the genus *Trichoderma* for the production of surfactants was assessed. At the same time, an attempt was made to pre-determine their chemical nature, depending on culture duration and conditions. 

## 2. Materials and Methods

In this study, the following strains of filamentous fungi were employed: *Trichoderma atroviride* SB6. *Trichoderma citrinoviride* HL, *Trichoderma citrinoviride* C1, *Trichoderma citrinoviride* B3, *Trichoderma citrinoviride* B11, *Trichoderma harzianum* T33 and *Trichoderma reesei* QM9414 (*Trichoderma reesei* Simmons (ATCC^®^ 26921^™^). The strains originated from own collections and were genetically identified to species on the basis of *ITSTest*-fungi [13]. 

Screening cultures were conducted on two types of mineral-based media with varied addition of a carbon source, Saunders (g/L)(KH_2_PO_4_—0.20; K_2_HPO_4_—0.15; NaH_2_PO_4_—2.00; Na_2_HPO_4_—1.50; NH_4_NO_3_—0.60; NaNO_3_—3.80; MgSO_4_—0.30; peptone—5.00; glucose 0 (S1), 10 (S2)) and Mineral Glucose Peptone (g/L)—(KH_2_PO_4_—2.00; MgSO_4_—1.00; CaCl_2_—1.00; (NH_4_)_2_SO_4_—3.00; peptone 5 (M1); peptone 2; glucose 10 (M2)), in 250 mL Elenmayer flasks containing 50 mL of medium. Screening cultures were performed in two replicates [14].

Culture fluids, after centrifugation (5000 rpm for 15 min), were subjected to following determinations (performed in triplicates):

Microwell screening test [15]: To a pure 96-well, flat-bottomed microplate, 150 μL of supernatant from each day of culture was added (2, 4, 6, 8, and 10-th). Distilled water was added as a control. A printed grid was placed beneath the plate. Along with decent biosurfactant properties for surface wetting, a visual distortion of the grid is observed.

Drop-collapse method [16]: The volume of 200 μL of distilled water, followed by 50 μL of olive oil and 40 μL of supernatant were added to the 96-well, flat-bottomed microwell plate. As control, 200 μL of distilled water was used. After 10 min, the drop shape and overcast zone were observed. This was an indicator of the presence or absence of surfactants. If biosurfactants are present in the test fluid, the drop of culture fluid is unstable and spreads throughout the well.

Oil-spreading method [15]: To a clean 96-well, flat-bottomed microplate, 200 μL of distilled water was added, then 50 μL of engine oil, forming a mono layer floating on water, and 40 μL of culture fluid supernatants. The resultant clear zones were observed.

Surface tension measurement [17]: Measurement of surface tension in culture supernatants was performed on a Krüss K6 manual tensiometer, according to a ring tear-off method (measuring the detachment force of the metal ring with specified diameter from the surface of the tested liquid) for supernatants from each flask culture. Distilled water was used as a control. 

Separation of biosurfactants [18]: The cultures were conducted for 10 days, centrifuged at 5000 rpm for 5 min. The resultant supernatant was acidified with 6 M HCl to pH 2 to precipitate biosurfactans from the solution and left overnight at 4 °C. The precipitate was separated with centrifugation (5000 rpm for 15 min).

Thin-layer chromatography: Visualization of active compounds was carried out using classic thin-layer chromatography (TLC). After freeze drying, the fluid was dissolved in a mixture of methanol and acetone. To the freeze-dried samples, 50 μL of water was added. The silica gel plate was applied 2-fold after 5 μL of the test medium. The substrate itself was used as a control. The plate was placed in a chromatographic chamber with a developing system (chloroform:methanol:acetic acid; 5:3:2). After development, plates were dried and subjected to the three different detection techniques: (1) The plate was placed in an iodine chamber for lipids detection; (2) the plate was sprayed with 0.25% ninhydrin solution in acetone and heated for 5 min at 105 °C in order to detect peptides, (3) the plate was sprayed with solution A (0.15 g orcin + 8.2 mL sulphuric acid 65% + 42 mL H_2_O) and heated for 10 min at 105 °C to detect sugars.

### The Identification of Secondary Metabolites

Gas chromatography: The sample obtained during separation biosurfactant (10 mg) was mixed with 2.5% sulfuric acid-methanol reagent (1 mL). After the reaction was quenched with 1 mL of water, the sample was extracted with n-hexane and injected into GC–MS (GCMS-QP2010 SE- Shimadzu, Japan) using Zebron ZB-FAME capillary column (30 m × 0.25 mm × 0.20 μm). Split injection mode was used for injecting the sample (1 μL at 250 °C) using helium (1 mL/min).

Fourier Transformation Infrared Spectrometry: The Fourier Transformation IR Spectrometry (FTIR) method was performed on IRSpirit spectrometer with QATR-S (SHIM-POL) with a measuring attachment for weakened multiple reflections of weakened diamond prism (ATR). The obtained spectra were recorded in the range of wavenumbers from 4000 cm^−1^ to 400 cm^−1^. FTIR analysis was carried out on the basis of the organic (methanol:acetone 3:2) extract and the cooled foam obtained from the final culture. 

Nuclear Magnetic Resonance: The chosen biosurfactants (BSs) were subjected to further analysis with NMR. The ^1^H spectra were measured at 298 K using a Bruker Avance 500 MHz spectrometer equipped with a 5 mm diameter tube containing 600 µL of 5 mg sample, with D_2_O as a solvent. The proton experiment was run using the zg30 pulse program. Chemical shifts are given in parts per million (ppm) to the signal of the solvent (D_2_O, 1H: 4.79 ppm) [19,20].

Statistical analysis: One-way analysis of variance (ANOVA) was applied along with Duncan’s test to determine statistically homogenic groups. Statistica 13 (TIBCO Software, Inc.) software was used.

## 3. Results

On the basis of preliminary screening tests, the strains that exhibited the highest potential for the production of surfactants were selected. In the microplate screening test, positive effects of extracellular metabolites were observed for strains *T. citrinoviride* HL and B3 from the 2-nd day of culture, as well as *T. citrinoviride* B11 and C1 from the 4th and 6th day of culture in Saunders medium. However, after a prolonged cultivation period (8 days), *T. citrinoviride* B11 and HL and *T. harzianum* T33 were dominant.

A positive result of the experiment was also determined in the culture fluid of the C1 strain from the 2-nd day of culture in MGP medium, as well as on culture days 4, 6, and 8 of the B11 and HL strains. Interestingly, the extension of the culturing time improved the results. From the 10th day, cultures of B11, HL, C1, and T33 strains in both, Saunders and MGP media, the most satisfactory effects were obtained.

Another test for the presence of biosurfactants in each of the post-culture fluids was the drop-collapse method. After application of the samples, the drop shape and transition zone were evaluated. Similar result was obtained in each of the tests, which indicated the presence of surfactants, especially high for strains B11, HL, C1, and T33 grown in both, Saunders and MGP medium.

The last of the optical tests was the oil-spreading technique. The image showing the explicit and large clearing zone in the wells of the microplate appeared in the post-culture fluids of the B11, HL, C1, and T33 strains (data not shown).

Examining the direct effect of extracellular products isolated from cultures of *Trichoderma* strains, a significant reduction in surface tension was denoted. The results, in relation to water and pure control substrate, revealed slight differences between producers. The *T. citrinoviride* C1 strain turned out to be the best producer of biosurfactants. In its culture, the lowest value of the surface tension (31.5 mN/m), was obtained in the Saunders 2 medium, containing higher amount of glucose, while the control was 61.5 mN/m. Conversely, the *T. reesei* QM9414 strain was the least effective biosurfactant producer among the tested microorganisms. In the systems where the post-culture fluid was exposed to TCA before the test, the surface tension was also reduced in the control sample and in distilled water, thus slightly disturbing the overall picture. Statistical analysis confirmed the higher ability to reduce surface tension by three of the tested strains, as well as a significant difference (a-f) to the control substrate and pure distilled water (Table 1).

The occurrence of individual components was determined with TLC. At first, the detection of biosurfactants’ lipid fraction was performed (Figure S1). Positive results were obtained after the culture in Saunders 1 medium, without glucose, and Saunders 2, supplemented with a higher concentration of glucose. An exceptionally clear result was observed in the case of the C1 strain. For the strains tested in the MGP 1 medium, low amounts of lipid parts were observed. Consecutively, the analysis was conducted towards the detection of peptidic constituents (Figure S2). It was found that none of the selected *Trichoderma* strains grown in enriched media produced components of peptide surfactants. For complete characteristics of the tested compounds, the solvent mixture was changed to one composed of chloroform and ethanol. At first, the detection of lipid parts in culture in enriched media was performed. The result was considered positive (Figure S3). All strains, except *T. harzianum* T33 in Saunders 2 medium, were capable to produce lipid components. In the performed TLC for the detection of proteins (Figure S4), a positive result was also found; thus, it was shown that selected *Trichoderma* strains had the ability to produce lipid and peptide surfactants.

Infrared spectroscopy (FTIR) was applied to characterize the global structure of *Trichoderma*-derived surfactants. Absorption signals were obtained for the following bonds: C-O, C=C, C=N, N-H, and O-H. In the Saunders 1 medium, the presence of certain functional groups was demonstrated in almost each of the post-culture fluids of the tested strains. In the extracellular products isolated in Saunders 2 medium, which contained a greater amount of glucose than in the basic composition, the obtained results were satisfactory. From the culture in MGP 1 medium without glucose, all of the previously mentioned functional groups, except C-O, were detected in each culture fluid. In the final test medium, MGP 2, where the glucose content was the highest, there were significant differences in the basic composition of spectra. In the control sample, i.e., the culture medium itself, only the presence of the C-O group was detected, which however was absent in the post-culture fluid of the B3 and B11 strains (Table 2). The analysis of products extracted with a mixture of chloroform and ethanol did not affect the absorption bands. Furthermore, foam formed on the surface of culture fluids after vigorous stirring was tested (Table 2). It was confirmed that all low m.w. compounds migrated to foam. 

The widest spectrum of the sought compounds was detected in the culture fluid of the B3 strain, separated from all production media. The characterization of the possible fatty acid units was carried out using GCMS. Clearly, there were some differences between the four tested media, as well as certain regularities. In Saunders 1 medium, the highest concentration of acids was obtained in the control sample, where the following units were present: 10:0, 14:0, 16:0, 18:0, 18:1cis and 20:0. In the culture fluids of the HL and B3 strains, there were extracellular products such as: 18:2trans, 18:3cis 9, 12, 15 and 20:4cis 5, 8, 11, 14.

The concentrations were slightly lower as compared to the control, within the range of 195.22–184.81 µg/mL. After application of another culture medium, Saunders 2, a large number of units and the highest concentration were recorded for the C1 strain (15:0, 16:0, 17:0, 18:0, 18:1cis, 18:2trans, 18:2cis, 20:3cis 8,11,14, 20:4cis 5,8,11,14 and 22:6cis 5,8,11,14,17). The MGP 1 medium exhibited the highest amount of fatty acids in the control sample. The concentration was as high as 702.838 µg/mL. In the cultivation of each strain, the amount of fatty acids was reduced and new units appeared. It was most evident in the SB6 culture fluid (Table 3).

The total content of the biosurfactant compounds in post-culture supernatants of the tested strains was evaluated. To achieve this, extraction with organic solvent mixture was performed, followed by drying of the obtained extract. Obviously, not only the compounds of interest, but also the accompanying compounds migrated into the solution. However, a certain m.w. profile was obtained that differentiated both the strains and, to a lower extent, the production media (Table 4).

The m.w. of the products transferred to the organic solvent in a few cases exceeded 1 g/L and in the vast majority of cases was 3–4 times lower than the residue in the aqueous phase. It is worth emphasizing that no correlation was found between the amount of compounds in the organic phase and the images of previously obtained TLC chromatograms.

**Figure 1:**
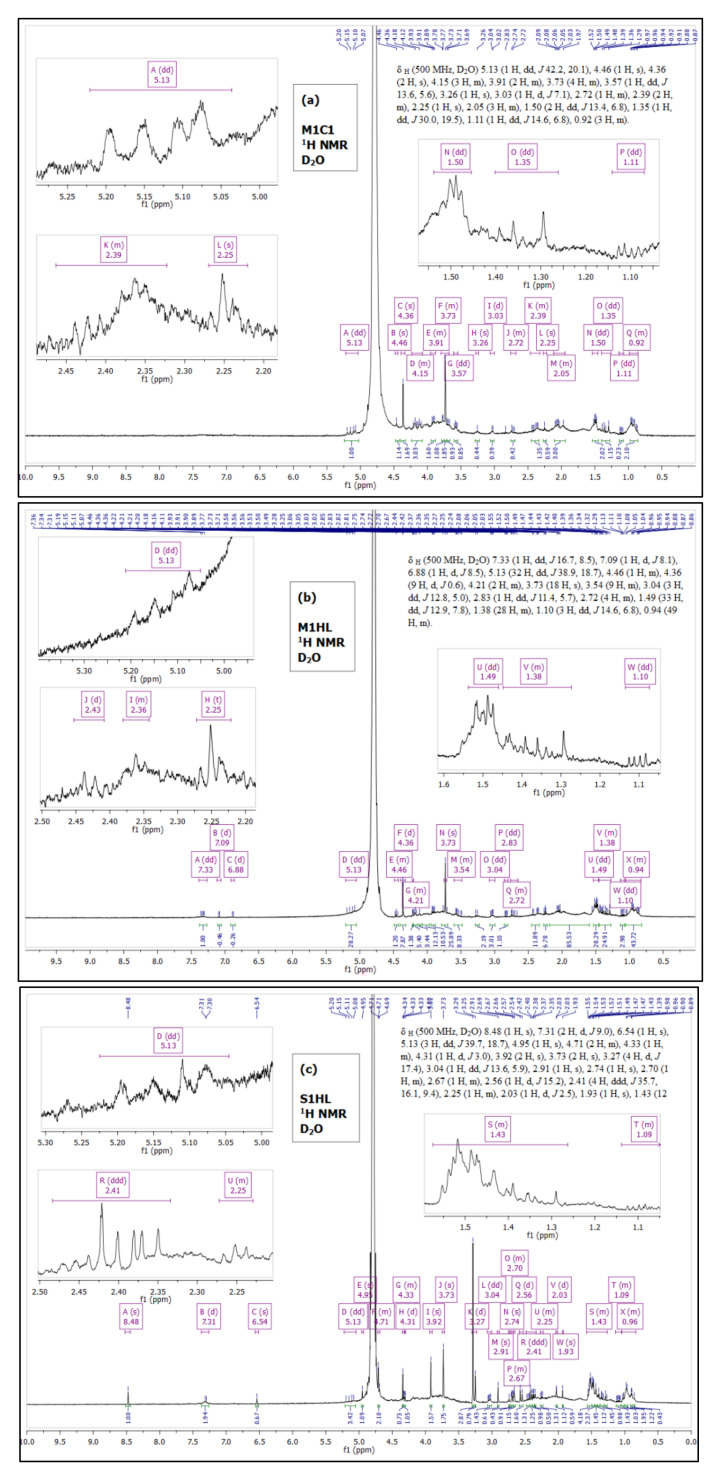
^1^H NMR spectra of BSs produced by C1 and HL strains of *Trichoderma citrinoviride* on the M1 (**a**,**b**) and S1 (**c**) substrates.

To confirm the data obtained with IR and GC, the samples were submitted to the analysis of ^1^H protons in NMR. Figure 1 presents ^1^H spectra of the biosurfactants produced by *Trichoderma* strain (C1 and HL) on the MGP M1 (Figure 1a,b) and the Saunders’ S1 (Figure 1c) substrates, respectively. A number of peaks is observed in NMR due to the presence of proteins and aliphatic fatty acids chains. Hence, a lipopeptide structure of the produced biosurfactant was confirmed. The proton NMR spectra appear complex; however, some distinguished peaks are present and their chemical shift (δ) was evaluated. Namely, for all samples, a chemical shift was recorded at: 5.25–5 ppm, corresponding to an olefinic hydrogens (H attached to =) in the fatty acid chain, 4.5–4 ppm—an aliphatic carbon-hydrogen bond, 2.2–1.8 ppm—allylic -CH_2_- (-CH_2_-C=) and at 1.1–0.8 ppm indicating –CH_3_.

Furthermore, the multiple chemical shifts at 1.15–1.08 ppm and 1.55–1.25 ppm (see insets in Figure 1) indicate the presence of long aliphatic chain (-CH_2_-)_n_. The analysis confirms also peaks at 3.9–3.5 ppm and 2.5–2.2 ppm corresponding to C attached to O representing methoxy group (-O-CH_3_) of the fatty acid.

However, certain bulbs and shoulders are present in the spectra, revealing some traces of impurities in all samples. Thus, a perfect integration during NMR analysis was not achieved. For biosurfactants produced by the HL strain (Figure 1b,c), additional weak signals of protons were detected above δ 6.5 ppm. It cannot be excluded that these chemical shifts represent amino acids that might be arranged in a cyclic structure. 

## 4. Discussion

Filamentous fungi of the genus *Trichoderma* are unique as they exhibit a multifarious enzymatic potential, and therefore receive worldwide attention. Their operation is mainly based on the abundant production of lytic enzymes, related to the parasitic abilities, antibiosis (the release of toxic chemicals that inhibit the development of other microorganisms), competition for nutrients and space with other microorganisms, the ability to modify environmental conditions, stimulating plant growth, inducing resistance in plants [7].

In the literature and other online resources, the production of surfactants by filamentous fungi, including the *Trichoderma* genus, is sparsely mentioned. For this reason, this topic seems intriguing and innovative. However, the selection of the designations was made on the basis of the literary entries of authors such as Chen et al. [21] and Youssef et al. [15], which mainly concerned bacteria and yeast. The authors who focused on the analysis of any fungal biosurfactants applied similar analytical methods to those presented in this work, as well as the procedure for metabolite separation. The drop-collapse method appears to be the simplest, however the results obtained are not only often misleading, but also dependent on the duration of the analysis [1,9,15,22]. Another simple and reasonably fast method is surface tension assessment. The obtained results allowed to identify potential producers of extracellular biosurfactants in a physical manner, without the need for any chemical reactions. The determined values for distilled water should normally be 72.8 mN/m [22]; the smaller those values, the higher the bioproducer potential is. It is also important to consider surface tension shift exerted by culture media alone. Many authors suggest that a reduction in the potential below 50 mN/m already qualifies the microorganism for further testing [22], while the level of 40 mN/m suggests a potential worth deeper analysis [15,23]. The best biosurfactant producers reduce surface tension to 30 mN/m or lower [24].

The production process in the presented study was carried out in submerged conditions in Saunders and MGP media, in basic composition, as well as after enrichment with peptone and glucose. Considering the carbon source, glucose and sucrose were as a rule used in the production of biosurfactants. Sena et al. [9] evaluated the secretion of biosurfactants in different strains of fungi throughout different sources of carbon and nitrogen, at a concentration of 20 g/L and 10g/L, respectively, as well as soybean oil, sucrose, cellobiose, xylose, yeast, meat, and malt extract. The authors did not use the mineral solution for their tests, and the results for *Trichoderma* strains did not encourage them to further study them. On the contrary, according to Batista et al. [17], the most favorable glucose concentration for bacteria was 20 g/L. For the tested *Trichoderma* strains, 10 g/L glucose concentration and extending the culture period to 10 days resulted in a positive effect in relation to shorter cultures in basic media. Especially with regard to Saunders medium, a significant decrease in surface tension was denoted in each of the post-culture fluids. The largest difference was found in the post-culture fluid of the HL strain. The lowest value of 31.5 mN/m was recorded in the supernatant of the C1 strain. In the basic version of the medium, which contained only 5 g/L glucose, optimal values of the surface tension were obtained, well within the range between 30 and 50 mN/m presented by Youssef et al. [15]. On this basis, it was found that the medium supplemented with glucose had a positive effect on the production of given compounds. However, in the second mineral medium, MGP, it was important to supply the nitrogen source in the form of peptone up to 5 g/L. This resulted in a noticeable decrease in surface tension in the post-culture fluid of the B11 strain, equal to 35.5 mN/m. The lowest value of the surface tension, 31.5 mN/m, was obtained in the culture of the C1 strain in Saunders 2 medium. Conversely, the highest value of 61.5 mN/m was observed in the extracellular products of the QM9414 strain secreted in MGP medium. 

In the report presented by Rufino et al. [25], a key factor in the successful biosurfactant production was the development of an economical process that involved cheap materials and ensured high yield. To increase the efficiency of microbial biosurfactant production, the author suggested investigating inexpensive substrates such as by-products or waste from the food industry, as they account for about 50% of the total cost of production. In the mentioned study, a cheap fermentation broth based on an industrial residue was successfully applied for the production of a lipopeptide by *Candida lipolytica*. The combination of soybean oil refinery residues with glutamic acid resulted in high production of biosurfactants, and therefore significantly reduced surface tension in the culture fluid. The surface tension dropped very quickly from approx. 50 mN/m to 25 mN/m in the early stages of growth, indicating excellent surface activity. In our study, the difference between the highest value of surface tension (61.5 mN/m in the Saunders 2 control medium) and the lowest value (31.5 mN/m in the culture of the C1 strain) was 30 mN/m. It was essential to modify the components of the culture medium, and to apply glucose more precisely. Santa Anna et al. [26] reached similar conclusions.

Biosurfactants produced by bacteria that are secreted as intracellular or extracellular metabolites during cell growth cause a significant reduction in surface tension. *Pseudomonas aeruginosa* is one of the extensively studied species in terms of biosurfactant production. Most of the biosurfactants produced by this bacterium caused a reduction in surface tension to a value of 27.28 mN/m. Similar studies for *Bacillus subtilis* MUV4 were also conducted by Suwansukho et al. [18]. The authors demonstrated the ability of the strain to produce biosurfactants. They determined that the surface tension of the culture medium decreased from 53.50 mN/m to 33.50 mN/m in the culture fluid after 48 h of cultivation. In our study, nearly identical minimal value of surface tension was achieved. This confirmed the great underestimated potential of the *Trichoderma* fungi, especially the C1 strain. Despite the use of different culture conditions than in the publications discussed above, the obtained results were equally satisfactory.

The potential for surfactant production of *Trichoderma* fungi was investigated using thin layer chromatography (TLC), which is a simple technique used to separate products in small quantities. The nature of the produced biosurfactants was assessed with the use of a suitable solvent (i.e., methanol + acetone and chloroform + ethanol). In the culture fluids of selected strains, mainly lipid parts of surfactants and lower amounts of protein were obtained. At this stage of research, it was not possible to define deeper conclusions. The same method was used by Batoll et al. [27] in studies of bacterial strains isolated from soil. The biosurfactant produced by the isolates was a lipid in structure as it produced yellow-brown spots upon contact with iodine with an Rf = 0.61 identical to that of rhamnose standard (Rf = 0.63). The researchers determined the glycolipid nature of the compounds and the presence of the rhamnose sugar.

Lyman et al. [28], examining potential of numerous microorganisms to reduce the surface tension in the growth medium, decided to test *Rhodotorula babjevae* MD1169 and *R. taiwanensis* MD1149 as potential candidates for the production of a new biosurfactant. In the conducted research, an immediate drop in surface tension (to 33.8 mN/m) was denoted right at the beginning of the cultivation. In addition, the samples were analyzed by gas chromatography-mass spectrometry (GC-MS) to confirm the presence of biosurfactants. The authors showed that the biosurfactants in the growth medium reached their maximum concentration on the fourth day. The analysis revealed that in particular the MD1149 strain produced glycolipids composed of sugar alcohols mannitol and arabitol, as well as the main components of fatty acids, including 3-hydroxy-stearic acid (C18:0), 3-hydroxypalmitic acid (C16:0), acid octadecene (C18:1, double bond in position 2). The conclusions from gas chromatography carried out for the purposes of this study were slightly different and more diverse than for yeast, certainly influenced by the different conditions of the tests and cultures. The highest concentration (322.97 µg/mL) and a wide range of bonds was determined in the post-culture fluid of the HL strain isolated in the MGP 2 medium. The presence of the most common extracellular products was mainly detected as: 14:0, 16:0, 18:0, 18:1cis, 18:2trans, 18:2cis, 20:0. In addition, Matussin et al. [29] analyzed the potential of the selected *Trichoderma* strain towards biosurfactants production in mineral medium with 40 g/L sucrose, but also 2% of crude oil content. These studies indicated the specific presence of hexadecenoic acid (16:0) and octadecenoic acid (18:0), which correlates with the presented results. Likewise, the FTIR analysis confirmed that the examined biosurfactants are glycolipids [29].

Despite the use of different microorganisms and culturing conditions in our studies, the confirmed presence of biosurfactants together with the type and content of fatty acids was in accordance with above-mentioned reports.

## 5. Conclusions

The presented study provides a comprehensive evaluation of *Trichoderma* species so far undescribed in terms of biosurfactant production capability. In addition, a variability found among tested strains was confirmed to be a species-dependent feature. A variety of techniques was applied to assess the occurrence of biosurfactants, highlighting the strengths and weaknesses of these methods. Biosynthesis of biosurfactants proceeded in nutrient-deficient media, pointing to their likely constitutive nature. The presented results deepen knowledge of *Trichoderma* fungi and allow to better understand the nature of their broad features focused on their ability to restrict the growth of other microorganisms, known as biocontrol.

## Figures and Tables

**Table 1:** Values of surface tension [mN/m] by the physical ring tear-off method for culture fluids obtained in culture of selected *Trichoderma* strains (*T. citrinoviride* HL, C1, B3, B11; *T. atroviride* SB6; *T. harzianum* T33 oraz *T. reesei* QM9414) cultured in following media: Saunders, Saunders + 6% TCA (S + 6% TCA), Saunders 1 (S1), Saunders 2 (S2) and MGP, MGP + 6% TCA, MGP 1, MGP 2. Superscript letters (^a–f^) at strain designations represent significant differences between strains.

Strain	Growth Medium							
	Saunders	S + 6%TCA	S 1	S 2	MGP	MGP + 6%TCA	MGP1	MGP2
HL ^a^	39.0	41.5	40.5	36.0	38.1	40.2	37.5	38.0
SB6 ^c^	47.4	47.2	54.5	51.5	45.0	44.0	49.0	47.0
B11 ^a^	38.0	42.0	40.0	38.0	40.5	41.9	35.5	37.0
C1 ^a^	37.5	39.2	33	31.5	36.5	39.1	36.5	38.5
B3 ^a^	38.2	41.0	39.5	37.0	39.9	41.5	36.5	37.5
T33 ^b^	42.5	42.0	40.5	43.5	43.3	43.1	44.0	47.0
QM9414 ^d^	51.2	47.5	-	-	61.5	55.0	-	-
Control ^e^	60.5	53.9	61.5	61.0	65.9	54.0	60.0	62.0
diH_2_O ^f^	72.8	59.1	71.2	71.2	72.8	59.1	70.0	70.0

**Table 2:** Infrared spectroscopy results—occurrence of signals at specified wavenumbers; cultures organic extract (q) and culture fluid foam (x) of *T. citrinoviride* HL, C1, B3, B11; *T. atroviride* SB6 and *T. harzianum* in Saunders (S1, S2) and MGP (M1, M2) media.

Absorbance Signal—Foam (x); Extract from Culture (q)																	
Medium	Strain	1050–1430 (C-O)	1500–1610/1600–1680 (C=C)	1500–1650 (C=N)	1500–1650 (N-H)	1600–1870 (C=O)	2200–2400 (C=N)	3000–3500 (O-H)	Medium	Strain	1050–1430 (C-O)	1500–1610/1600-1680 (C=C)	1500–1650 (C=N)	1500–1650 (N-H)	1600–1870 (C=O)	2200–2400 (C=N)	3000–3500 (O-H)
S1	HL	q	q x	q x	q x			q x	S1	C1	q	q	q	q			q
S2		q x	q	q	q		x	q	S2		q x	q	q	q		x	q x
M1		x	q	q	q			q	M1		q x	q	q	q	x	x	q x
M2		q	q	q	q			q	M2		q	q	q	q			q
S1	SB6	q x	q	q	q			q	S1	B3	q	q x	q x	q x			q x
S2		q x	q	q	q		x	q	S2		q x	q	q	q		x	q
M1		x	q	q	q			q	M1		x	q	q	q	x	x	q x
M2		q x	q	q	q	X		q	M2			q	q	q	x		q
S1	B11	q	q	q	q			q	S1	K	q	q	q	q			q
S2		q x	q	q	q		x	x	S2		q	q	q	q			q
M1			q	q	q			q	M1			q	q	q			q
M2			q	q	q	X		q	M2			q	q	q			q

**Table 3:** Result of gas chromatography—types of bonds and amounts of fatty acids (µg/mL); cultures of *T. citrinoviride* HL, C1, B3, B11; *T. atroviride* SB6, *T. reesei* QM9414 and *T. harzianum* T33 in Saunders (S1, S2) and MGP (M1, M2) media.

	14;0	15;0	15;1	16;0	16;1	17;0	17;1	18;0	18;1	18;1 cis	18;2 trans	18;2 cis	18;3 cis 9,12,15	20;1 cis	20;3 cis 8,11,14	20;4 cis 5,8,11,14	22;1 cis13	22;2 cis-13,16	23;0	24;0	24;1 cis 15	22;6 cis 5,8,11,14,17	SUM
ug/mL																							
**S1/HL**				45				43	36		25	23	23										**195**
**S2/HL**				45				41	36	29	25	23											**198**
**M1/HL**			13	45	15				34		23						28						**159**
**M/HL**				49	18			42	43	32	26	24					27		30			32	**323**
**S1/SB6**				45				42															**86**
**S2/SB6**				46	16			41	37	29	24	23											**216**
**M1/SB6**	27	16	14	46	15		19	41											30			31	**240**
**M2/SB6**		16		47	17			41			24	23											**168**
**S1/B11**	26			45				43		30													**144**
**S2/B11**		16		46		21		42		30	26	24									24		**229**
**M1/B11**				45				41	35	29							36	33			27	32	**278**
**M2/B11**				45	15			41		28							27				25		**181**
**S1/C1**		16		45				42															**102**
**S2/C1**		16				21		41		28	23	22			27	25						31	**234**
**M1/C1**				45				41	36	28				20		27	27						**225**
**M2/C1**			12	46	15		19	41		28		22					27						**210**
**S1/B3**								41	34							27				63			**165**
**S2/B3**				45				41			24	23											**133**
**M1/B3**				45													30	32					**106**
**M2/B3**				45													27						**72**
**S2/T33**				45	15			41			24	23	24			27							**199**
**M1/T33**				45									23				27	32			25		**152**
**M2/T33**		16		45	16			41			23	22											**163**

**Table 4:** Dry matter extracted with organic solvent (including surfactants) and residue in the aqueous phase in g/L. Compilation of MGP (M1, M2) and Saunders (S1, S2) media.

Organic Solvent Extract in Different Production Media				
	**M1**	**M2**	**S1**	**S2**
K	0	0	0	0
HL	0.350	1.147	1.709	0.321
SB6	0.468	0.306	0.345	1.987
B11	1.787	1.240	0.992	1.678
C1	0.462	0.593	1.458	0.397
B3	1.011	0.476	0.106	1.595
T33	0.184	0.255	0.210	0.221
**Water Residues in Different Production Media**				
	**M1**	**M2**	**S1**	**S2**
K	0	0	0	0
HL	1.405	3.484	1.139	4.239
SB6	6.900	3.624	2.684	2.474
B11	3.213	1.847	4.459	5.092
C1	7.350	2.027	2.868	3.074
B3	0.286	7.750	0.789	5.158
T33	4.527	3.726	3.984	2.294

## Data Availability

The data presented in this study are available on request from the corresponding author.

## Supplementary Materials

The following are available online at https://www.mdpi.com/article/10.3390/biom11040519/s1, Figure S1: Visualization of the result of thin-layer chromatography (lipid detection) in a post-culture fluid dissolved in methanol and acetone of selected *Trichoderma* strains (*T. citrinoviride* B11, B3, HL, C1; *T. atroviride* SB6 and *T. harzianum* T33)—Saunders medium (S) and MGP (M1/M2). Figure S2: Visualization of the result of thin-layer chromatography (detection of peptides) in a culture fluid dissolved in methanol and acetone of selected *Trichoderma* strains (*T. citrinoviride* B11, B3, HL, C1; *T. atroviride* SB6 and *T. harzianum* T33)—modified Saunders (S1/S2) and MGP (M1/M2) media. Figure S3: Visualization of the result of thin-layer chromatography (lipid detection) in the culture fluid dissolved in chloroform and ethanol of selected *Trichoderma* strains (*T. citrinoviride* B11, B3, HL, C1; *T. atroviride* SB6 and *T. harzianum* T33)—modified Saunders (S1/S2) and MGP (M1/M2) media. Figure S4: Visualization of the result of thin-layer chromatography (detection of peptides) in the culture fluid dissolved in chloroform and ethanol of selected *Trichoderma* strains (*T. citrinoviride* B11, B3, HL, C1; *T. atroviride* SB6 and *T. harzianum* T33)—modified Saunders (S1/S2) and MGP (M1/M2) media.
