# Supplementary material for: Biosurfactants from Trichoderma Filamentous Fungi—A Preliminary Study
Source: Biomolecules. 2021 Mar 30;11(4):519. doi: 10.3390/biom11040519 (PMC8067329; doi:10.3390/biom11040519)
Supplement: Supplementary file 1 [file biomolecules-11-00519-s001.pdf]

biomolecules

MDPI

Article

Biosurfactants from *Trichoderma* Filamentous Fungi—A Preliminary Study

Piegza Michał [1,*], Pietrzykowska Joanna [1], Trojan-Piegza Joanna [2] and Łaba Wojciech [1]

[1] Department of Biotechnology and Food Microbiology, Wroclaw University of Environmental and Life Sciences, 37 Chelmonskiego Street, 51-630 Wroclaw, Poland; joanna.pietrzykowska@o2.pl (P.J.); wojciech.laba@upwr.edu.pl (Ł.W.)

[2] Faculty of Chemistry, University of Wroclaw, 14 F. Joliot-Curie Street, 50-383 Wroclaw, Poland; joanna.trojan-piegza@chem.uni.wroc.pl

* Correspondence: michal.piegza@upwr.edu.pl

Citation: Michał, P.; Joanna, P.; Joanna, T.-P.; Wojciech, Ł. Biosurfactants from *Trichoderma* Filamentous Fungi—A Preliminary Study. *Biomolecules* **2021**, *11*, 519. https://doi.org/10.3390/biom11040519

Received: 10 February 2021
Accepted: 26 March 2021
Published: 30 March 2021

Publisher's Note: MDPI stays neutral with regard to jurisdictional claims in published maps and institutional affiliations.

Copyright: © 2021 by the authors. Submitted for possible open access publication under the terms and conditions of the Creative Commons Attribution (CC BY) license (http://creativecommons.org/licenses/by/4.0/).

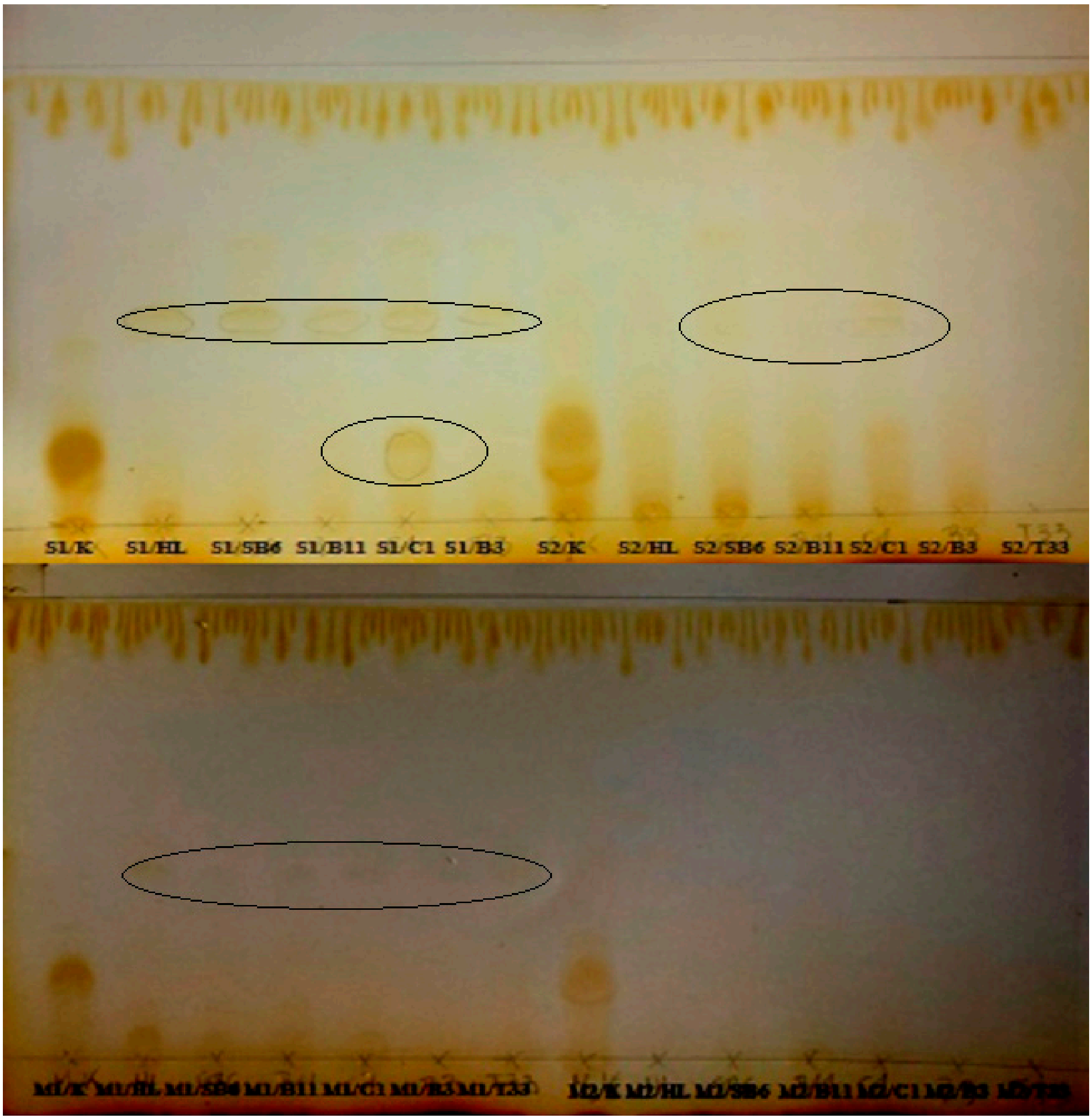

Figure S1. Visualisation of the result of thin-layer chromatography (lipid detection) in a post-culture fluid dissolved in methanol and acetone of selected *Trichoderma* strains (*T. citrinoviride* B11.B3.HL.C1; *T. atroviride* SB6 and *T. harzianum* T33) - Saunders medium (S) and MGP (M1/M2).

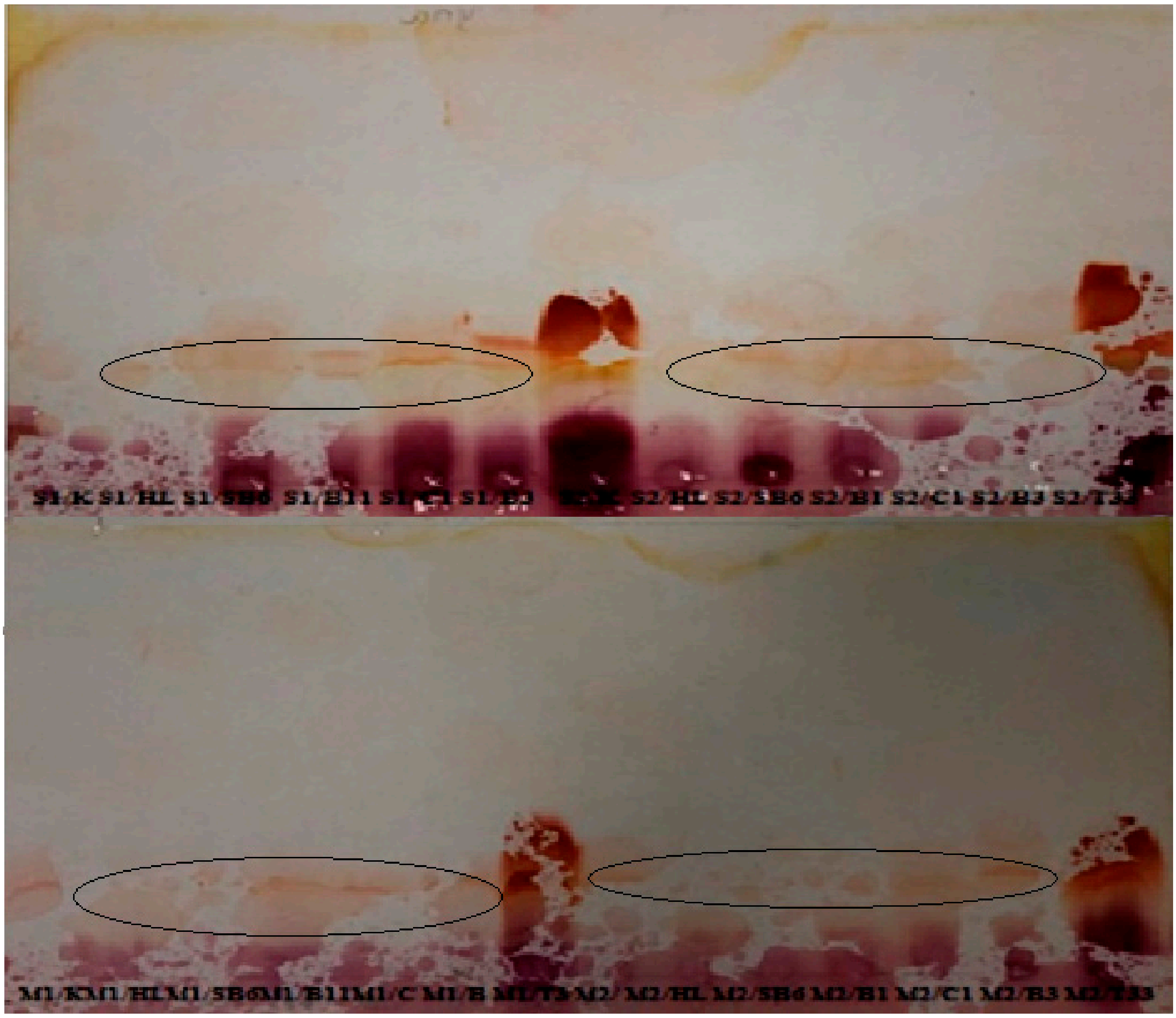

Figure S2. Visualization of the result of thin-layer chromatography (detection of peptides) in a culture fluid dissolved in methanol and acetone of selected *Trichoderma* strains (*T. citrinoviride* B11.B3.HL.C1; *T. atroviride* SB6 and *T. harzianum* T33) - modified Saunders (S1/S2) and MGP (M1/M2) media.

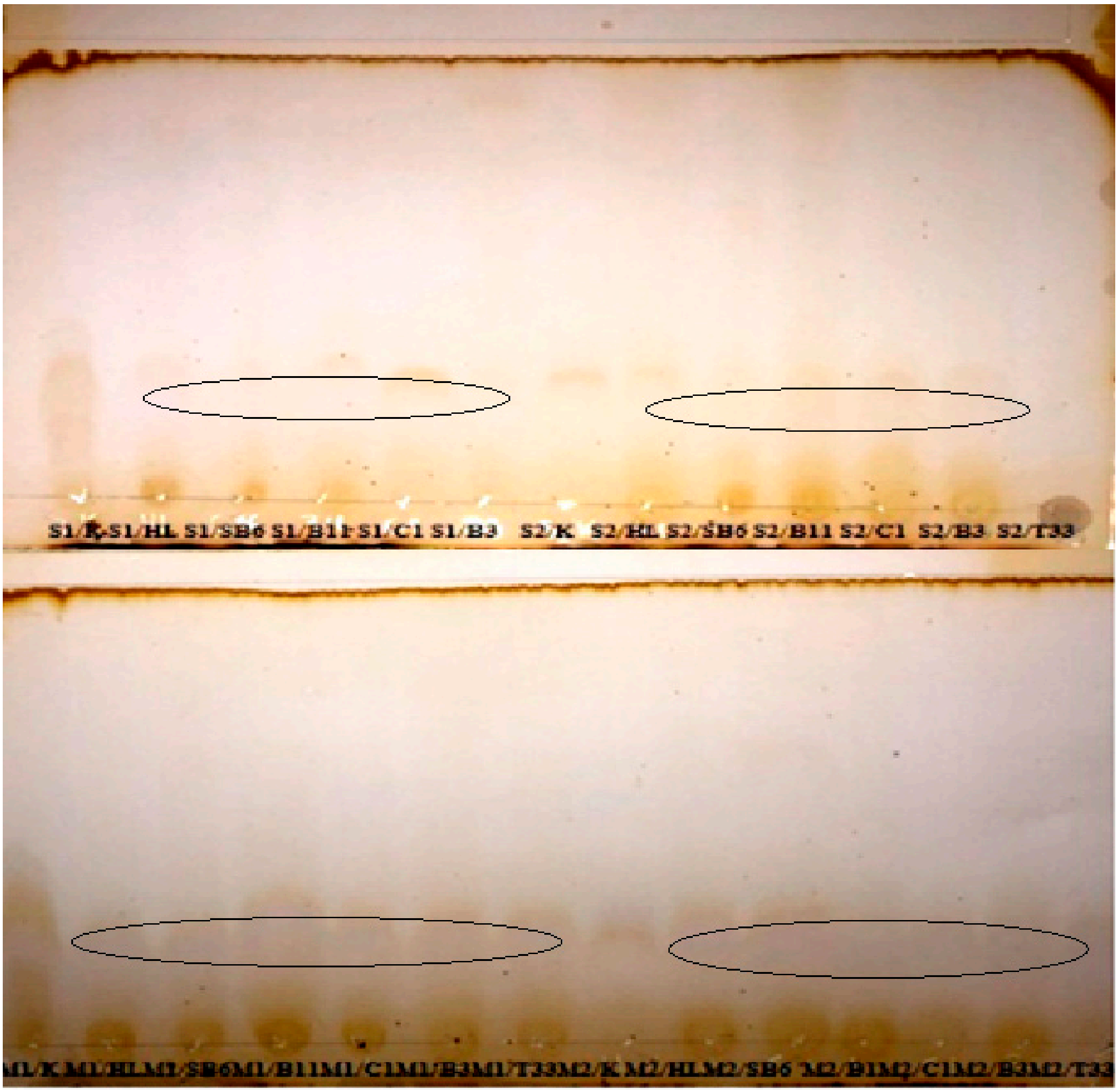

Figure S3. Visualization of the result of thin-layer chromatography (lipid detection) in the culture fluid dissolved in chloroform and ethanol of selected *Trichoderma* strains (*T. citrinoviride* B11.B3.HL.C1; *T. atroviride* SB6 and *T. harzianum* T33) - modified Saunders (S1/S2) and MGP (M1/M2) media.

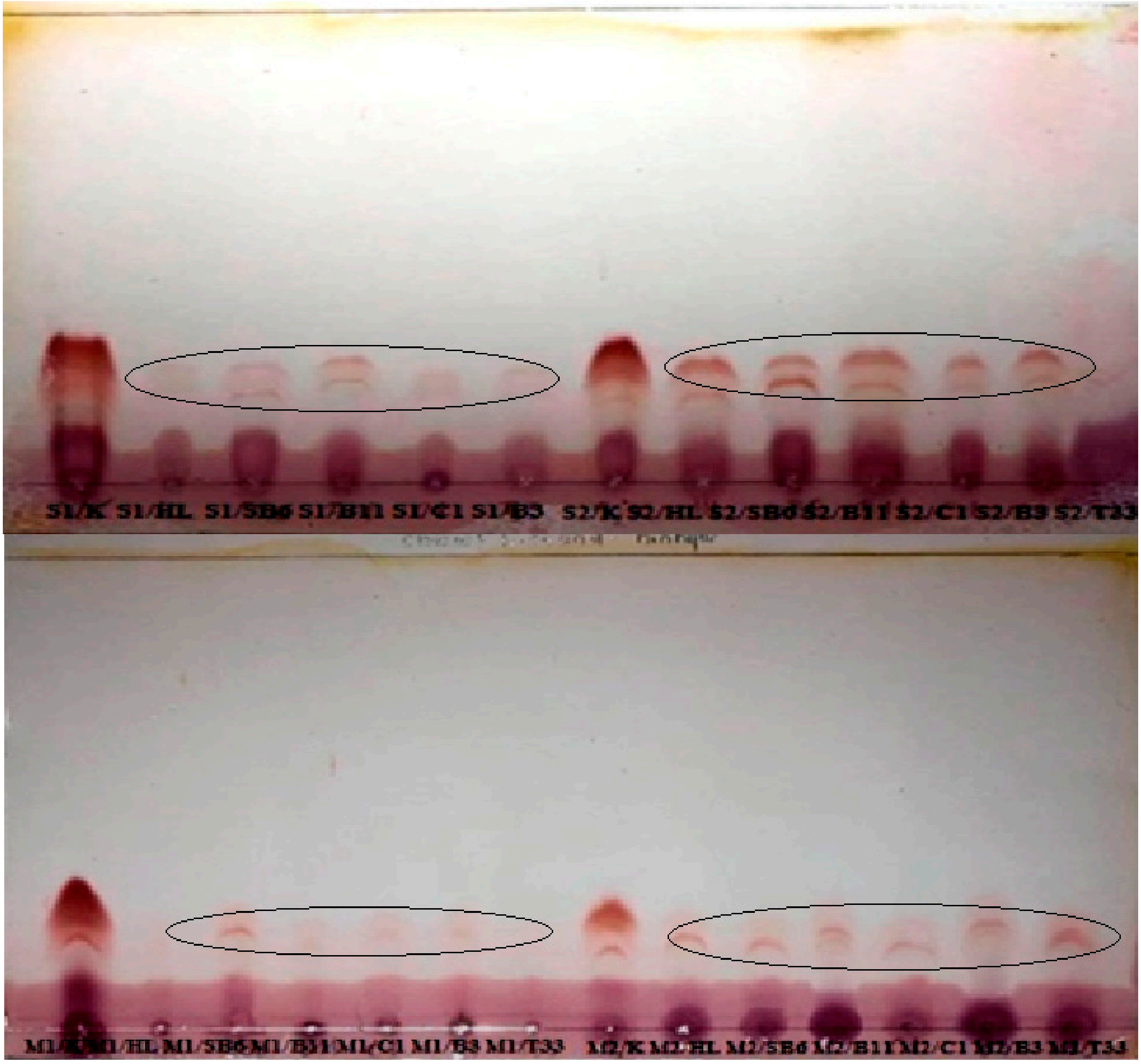

Figure S4. Visualization of the result of thin-layer chromatography (detection of peptides) in the culture fluid dissolved in chloroform and ethanol of selected *Trichoderma* strains (*T. citrinoviride* B11.B3.HL.C1; *T. atroviride* SB6 and *T. harzianum* T33) - modified Saunders (S1/S2) and MGP (M1/M2) media.
